# Quantitative and qualitative analysis of the effects of human-animal interactions on health and well-being of older adults: an umbrella review

**DOI:** 10.1186/s12877-025-06487-3

**Published:** 2026-01-22

**Authors:** Giulia Grotto, Alessandra Buja

**Affiliations:** 1 Department of Pharmaceutical and Pharmacological Sciences, University of Padua, Via Marzolo, 5, Padua, 35131 Italy; 2 Department of Cardiological, Thoracic and Vascular Sciences, and Public Health, University of Padua, Via Loredan, 18, Padua, 35127 Italy

**Keywords:** Human-animal interactions, Pets, Older adults, Emotional health, Mental health, Physical health, Well-being

## Abstract

**Background:**

Research shows that older adults can experience physical, emotional, and social benefits from owning or interacting with pets. Different variables, such as the type of animal, the context, and the duration of human-animal contact, can influence the effects of animal companionship on health. This umbrella review aims to assess how interactions between animals and older adults impact health and wellbeing.

**Methods:**

After the definition and registration of the review protocol, five electronic medical databases (PubMed, PsycINFO, Scopus, Web of Science and Cochrane Library) were systematically searched in June 2024 to find relevant second-level studies. Systematic reviews or meta-analyses assessing the association between exposure to animals and the physical, emotional, and mental health and well-being of individuals aged 60 years or older were included; studies focusing exclusively on animal-assisted treatments, conducted on older adults with a specific disease or medical condition, and written in a language other than English were excluded. The extracted information comprised the main author and year, objectives, review type, participant details, context and setting, number of databases searched with date range, number and design of studies included, quality ratings, methods used to synthesize evidence, and outcomes. The data extracted from qualitative and quantitative research were presented according to the different types of exposure and summarized for the different outcomes using a tabular presentation.

**Results:**

The main findings regard the beneficial effects of dog-assisted interventions on social interaction and loneliness of older adults living in facilities; also, dog owners appeared to engage in high levels of walking activity. Qualitative research reported that older adults attributed to pets an important role and a variety of beneficial impacts (i.e., emotional support, sensory engagement, reciprocal interactions); some negative aspects related to pet ownership were also described as worries for pets’ health, and pets’ related expenses.

**Conclusions:**

Older adults living in facilities could benefit from dog assisted interventions to improve social interactions and decrease loneliness. Dog owners are found to benefit from the animal’s companionship, particularly regarding higher levels of physical activity due to dog walking.

## Introduction

Many nations across the globe are experiencing a rise in life expectancy, leading to a concurrent increase in the older population afflicted by chronic conditions that impair both physical and psychological well-being, thereby restricting their social engagement [1, 2].

Research suggests that owning or interacting with a pet, can provide physical, emotional, and social benefits for older adults [3]. For instance, human-animal interactions are associated with reduced depression, loneliness, and anxiety, and with improved quality of life, physical activity, and social connections [4, 5].

Over the years, research on human-animal interactions has developed and a variety of terms have emerged to describe the many contexts in which human-animal interactions can occur, particularly with regard to the range of services involving animals benefiting people [6].

Human-animal interactions refer to an umbrella concept described as “any manner of relationship or behavior between people and animal(s). These interactions can vary widely and be positive, negative, or neutral for either party and can occur in individual, community, or societal contexts” [7]. Of all possible human-animal interactions, Animal Assisted Services (AAS) identify “the full spectrum of practices in which animals are included in various roles for the benefit of humans” [6] and include three different categories: animal-assisted treatment (AATx), animal-assisted support programs (AASP), and animal-assisted education (AAE) [6]. While AATx imply the integration of animals as a critical component of a mental or physical health professional treatment, AASP includes “programs in which animals are engaged, directly or indirectly, in activities aimed at supporting and enhancing the well-being of humans” [6]. These programs do not focus on the treatment of a specific disease or clinical condition and may have aims that include increased motivation, prevention of loneliness and isolation, reduction of tension and anxiety, distraction from difficult situations, or emotional comfort [6]. Finally, AAE refers to “any educational program in which animals are integrated, directly or indirectly, as a critical component of an ongoing educational process” with the goals of promoting personal or organizational development in several possible areas (i.e., emotional regulation, coping strategies, prosocial skills, and/or empathy development) [6].

Concerning AASP, studies conducted in older adults living in care facilities have identified them as one complementary method of support that offers a wide range of benefits on well-being [8], specifically in improving psychosocial and physiological functioning [9]. The research outcomes indicate that AASP might promote a sense of connection among senior residents, relatives, and staff members within a care living facility, which is a key aspect of effective care [10].

As research on senior pet owners shows, exposure to a pet seems to benefit also independent older people living in the community [5] and, in general, the benefits associated with pet ownership or exposure to pets in various types of settings, and as an augmentation to traditional therapy are starting to be realized [11–13].

However, the results of studies on the effects of AASP do not provide consistent outcomes [14–16], and living with animals could also have negative effects, such as increased risk of falls, allergies, transmission of diseases, psychological dependency, and excessive grief responses after pet bereavement [17–19].

The health impact of exposure to animals might depend on many variables such as the type of animal, the context, the characteristics and the duration of the human-animal interaction [15]. In fact, the exposure of a pet-owner to his pet is qualitatively and quantitatively different from that between an older adult living in a nursing home and the animals visiting the facility during an AASP. Currently, most reviews rely on results from different types of AASP or studies on pet ownership, with substantial heterogeneity across the inclusions of populations and animals [15]. Given the significant variation across settings and human-animal interactions, these reviews provide little relevance for the understanding of the effects of animal companionship on health and wellbeing of older adults. The aim of this umbrella review was to assess the effects on the health and wellbeing of older adults resulting from different types of exposure and interactions that may exist between animals and older adults, particularly in the context of AASP for seniors living in care facilities and pet ownership for community-dwelling older adults.

## Methods

This umbrella review was carried out through a critical appraisal of the existing literature, beginning with a synthesis of previously published systematic reviews and evaluating the overall body of evidence. The research method was defined in advance and conformed to the Preferred Reporting Items for Systematic Reviews and Meta-Analyses (PRISMA) guidelines [20], which are thoroughly presented in the PRISMA checklist (Appendix 1). The review protocol was prospectively registered in the International Prospective Register of Systematic Reviews (PROSPERO – CRD42024557581). In order to examine the effects of human-animal interactions on the health and well-being of older adults in relation to different real-life experiences, a mixed-method approach was adopted, which includes the analysis of both quantitative and qualitative second-level studies [21]. Incorporating qualitative reviews offers a more holistic method to understand the impacts of older adults’ exposure to animals on their health, considering some of the difficulties in its evaluation. In particular, many of the effects that can result from human-animal interaction are qualitative in nature (e.g. perceived companionship, emotional support, reduction in loneliness) and may not be fully captured by quantitative metrics. In light of these challenges, incorporating qualitative data allows for a richer and more nuanced understanding of older people’s experiences with animals, complementing quantitative findings and providing a context that can guide future research and interventions.

### Search strategy

For the present study, the PubMed, PsycINFO, Scopus, Web of Science and Cochrane Library databases were systematically searched in June 2024. The choice of these specific databases was guided by the aim of being as comprehensive as possible to include all reviews pertaining to the research area; the criteria used for the choice were the specificity of the database (in the biomedical and psychological fields), the breadth and coverage of the database.

The search strategies were formulated by integrating keywords and their respective synonyms, which pertained to the three core domains of the investigation—‘older adults’, ‘health’, and ‘animals’—using Boolean operators. The search conducted in PubMed incorporated both MeSH terminology and unstructured keywords. Complete search methodologies can be found within Appendix 2.

Study selection was carried out independently by two authors (GG and AB) through a two-stage screening process: first by reviewing titles and abstracts, followed by full-text screening to assess eligibility. Disagreements between the two reviewers were resolved through discussion, and collective decisions were made. Reference lists of the included studies were also screened to identify any additional relevant publications.

### Inclusion and exclusion criteria

The questions guiding the review were established in advance, employing the PICO framework (Population, Intervention, Comparison, Outcome) for quantitative data and the PICo model (Population, phenomena of Interest, Context) for qualitative investigations [22, 23]. The population group was identified in older adults, the interventions were related to the exposure to animals, the comparison was identified in non-exposed older adults, and the outcomes were the impacts of the exposure on well-being, mental, emotional, or physical health. The phenomenon of interest for qualitative research was the perspectives of older adults experiencing animal companionship and, in the case of AASP in living facilities, also the experiences of the staff members; the context referred to the place where interaction with the animal occurred without restriction (i.e., both reviews focusing on community-dwelling older adults and on older adults living in residential facilities were included).

Reviews were considered for inclusion in the umbrella review if they:

- evaluated the association between the exposure to animals and the physical, emotional, mental health, and well-being of older adults;
- included individuals who were 60 years old and older;
- were written in English.

Reviews were excluded from the umbrella review if they:

- focused exclusively on AATx;
- were conducted exclusively on older adults affected by a specific disease or medical condition;
- incorporated theoretical studies or published opinions as their primary source of evidence;
- had a study design other than systematic review or meta-analysis (i.e. scoping review).

### Data extraction and data analysis

A data extraction form tailored to the research question was developed using Microsoft Excel. From each included study, the following information was extracted: (a) main author and year, (b) objectives, (c) type of review, (d) participants details, (e) setting and context, (f) number of databases sourced and searched, (g) date range of database searching, (h) publication date range, (i) number of studies and design, (j) instruments used to appraise the primary studies and the rating of their quality, (k) method of synthesis/analysis employed to synthesize the evidence, (l) outcomes. A descriptive analysis was performed to report the characteristics of the included reviews (Table 1) and their outcomes (Table 2). In addition, data from primary studies was characterized according to the different types of exposure (dog-assisted support programs, AASP, dog ownership, cat ownership, pet ownership) and synthesized for the various outcomes of interest (depression, apathy, anxiety, dementia/cognitive function, agitation, daily functionality, quality of life, loneliness, social interaction, general physical health, cardiovascular mortality, balance/falls, frailty, daily circadian cortisol cycle, nutrition and body weight, blood pressure, heart rate variability, physical activity), using a tabular presentation (Tables 3 and 4).

### Quality assessment of the included reviews

The methodological quality of the included systematic reviews was assessed using the AMSTAR 2 tool [153]. (Appendix 3). AMSTAR-2 categorizes systematic reviews into four quality levels—critically low, low, moderate, and high—by evaluating deficiencies across seven essential domains, rather than generating a numerical score. The two authors independently assessed the quality of the systematic reviews and disagreements were addressed through discussion until a consensus was reached.

## Result

### Identified studies

**Fig. 1:**
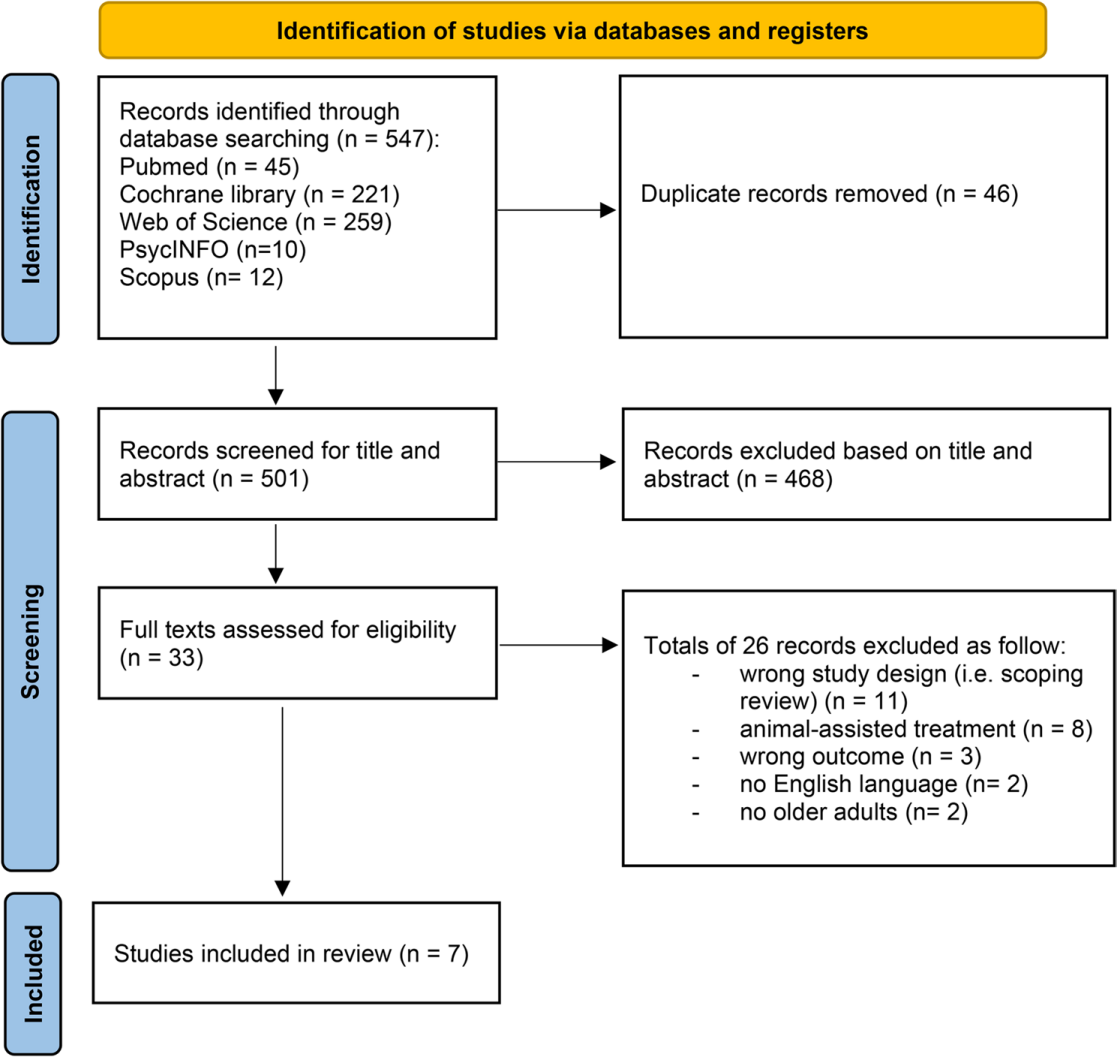
The preferred reporting items for systematic reviews and meta-analyses (PRISMA) diagram of the article search

The preliminary search generated 547 citations sourced from the electronic databases. Following the removal of duplicates, 501 titles and abstracts underwent screening, and 33 complete texts were evaluated for suitability. From these, 26 studies were omitted for the following reasons: they were not systematic reviews or meta-analyses (i.e., scoping reviews, n. 11 studies), they were focused exclusively on AATx (eight studies), their results did not concern the health or well-being of older adults (n. three studies), they were written in a language other than English (n. two studies), and they were not conducted on older adults (n. two studies). Ultimately, seven systematic reviews met the inclusion criteria. A manual search of the reference lists of the included reviews did not identify any additional studies of interest. The literature search is shown in detail in Fig. 1.

### Characteristics of the included systematic reviews

The characteristics of the included reviews and their outcomes are reported in Tables 1 and 2, respectively. All the included systematic reviews were recently published (from 2020 onwards); they collected a total of 143 primary studies, with an overall publication date range from 1982 [26] to 2021 [26, 27].

Four of the included reviews focused on community-dwelling older adults living with their pets [24, 25, 27, 28], while the other three focused on AASP conducted in care living facilities [5, 10, 26]. Four reviews included exclusively quantitative studies [5, 24, 25, 28], two included both quantitative and qualitative studies [10, 26] and one included exclusively qualitative studies [27]. Three of them included only studies on dogs [5, 10, 25], while the others also considered other types of animals, particularly cats, rabbits, birds, and fish. One systematic review was exclusively focused on mental health outcomes [25] while the others considered a wide variety of outcomes concerning both mental and physical health.

**Table 1:** Characteristics of the included systematic reviews

Main author, year	Objectives	Type of review	Participant details	Setting and context	Number of databases sourced and searched	Date range of database searching	Publication date range
Hughes et al., 2020 [5]	To investigate the effect of companion animals (whether simply as pets or used in more formal intervention approaches) on the physical and mental health of older adults	Systematic review	Older adults aged 60 years or above	Animal assisted interventions conducted in living facilities and in the community and pet ownership	2 databases (PsycINFO and PubMed)	From 2000 onwards	2000 - 2018
Jain et al., 2020 [10]	To review studies on dog-assisted interventions among older people in residential long-term care facilities and to provide an overview of their interventions, outcomes and methodological quality	Systematic review and meta-analysis	Older adults (65 years or more) residing in long-term care facilities. Pooled mean age: 83 years.	Facilities including nursing homes (n = 18), long-term care (LTC) facilities (n = 13) and other settings (n = 12) including specialized dementia care units, assisted living facilities and residential aged care services	18 databases (MEDLINE, EMBASE, PsycINFO, CINAHL, British Education Index, AMED, Social Policy and Practice, Web of Science, Social Care Online, SCOPUS, ERIC, ProQuest, International Bibliography of the Social Sciences, Science Citation Index, Cochrane Library, PubMed, Google Scholar and Open Grey)	January 2000 - December 2019	2000 - 2019
Kojima eta al., 2020 [24]	To identify evidence on associations between pet ownership and frailty among community-dwelling older adults	Systematic review	Community dwelling older adults with a mean age of 60 years or above	Community-dwelling older adults living with their pets	2 databases (PubMed and Google Scholar)	2000 - April 2020	2018 - 2019
Maurice et al., 2022 [25]	To establish whether owning a dog was associated with a lower number of symptoms of anxiety or depression and sleep disorders in community‐dwelling older adults.	Systematic review	Independent community-dwelling older adults, mean age over 70 years	Community-dwelling older adults living with their pets	8 databases (PubMed, EMBASE, Cochrane Library, Google Scholar, PsycINFO, Web of Science, OpenGrey and Grey Literature databases), plus a list of French MD theses	January 2005 - December 2020	2008 - 2019
Orr et al., 2023 [26]	To create a state-of-knowledge synthesis on the impact of animal-human interaction on care home residents and care home staff.	Systematic review and meta-analysis	Older adults residing in care homes perspectives or staff members' experiences of animals living at, or visiting, care homes.	Care homes (residential care home, nursing home, and long-term care facility)	14 databases (MEDLINE, Embase, APA PsycINFO, SPP, CINAHL Complete, AgeLine, CDSR, CENTRAL, DARE, ASSIA, Web of Science Core Collection, SCOPUS, ProQuest Dissertations and Thesis Global)	Up to July 2020	1982 - 2021
Reniers et al., 2022 [27]	To study the role and significance of pets for older adults	Systematic qualitative review	340 older adults (n= 115 males, n= 225 females); mean age 65 years and older.	Community-dwelling older adults living with their pets	2 databases (PubMed, PsycINFO), screening of reference lists of systematic reviews and searched HABRI central (an indexed specialized in human-animal interaction literature)	No limitation	2000-2021
Scoresby et al., 2021 [28]	To describe the body of knowledge surrounding the relationship between pet ownership and mental health.	Systematic review	The review considered the general population and then performed sub analysis. Only older adults were considered in the umbrella review.	Older adults living with their pets (cats or dogs)	2 databases (PubMed and Web of Science)	N.A.	1990 - 2020

*N.A. *Not Available

**Table 2:** Outcomes of the included reviews

Main author, year	Number of studies and design	Instruments used to appraise the primary studies and the rating of their quality	Method of synthesis/analysis employed to synthesize the evidence	Outcomes
Hughes et al., 2020 [5]	70 articles (animal-assisted intervention clinical trials, cross-sectional and longitudinal correlational surveys)	N.A.	Tabular and thematic analysis	In 52 out of 70 studies, companion animals had positive effects on the older adults. 29 studies focused on mental health and 23 of them identified positive outcomes especially regarding quality of life, depression, and the behavioral and psychiatric symptoms of dementia. 25 studies focused on physical health, and 85% of them identified positive benefits of companion animals, particularly in increases in physical exercise. 16 of the studies measured both mental and physical health variables and 7 of them found solely positive effects. Overall, 5 studies found that companion animals were only associated with negative effects, 4 studies found no effects, and 10 studies found equivocal results between the considered variables
Jain et al., 2020 [10]	43 studies (16 randomized control trials, 14 pre-/post design studies, 9 quasi-experimental studies and 4 qualitative cross-sectional studies)	Mixed methods appraisal tool (MMAT) criteria Most quantitative studies were assessed as low-quality (*n* = 26, 67%)	Thematic synthesis for qualitative studies, descriptive statistics for quantitative studies, random effects meta-analyses for RCTs where possible	Almost half of the quantitative studies (*n* = 18, 46%) found no significant changes over time or between groups across outcomes measured. 21 studies found positive effects, regarding the following outcomes: social functioning (*n* = 10), reduced depression (*n* = 6), and reduced loneliness (*n* = 5). A random-effects meta-analysis revealed a medium effect on reducing depressive or loneliness symptoms (pooled SMD: 0.66, 95%CI 0.21–1.11; I2 = 50.5; five trials), relative to treatment as usual. Key themes from qualitative studies included (a) animals as effective transitional objects to supplement missing interaction by either ‘filling a void’ or supplementing other human interactions and bonds (b) the therapeutic value of pets in reducing stress, spiritual connection, being in the moment, and ‘creating a good moment’, sensory comfort (c) the significance of the care environment and stakeholders in facilitating dog-assisted interventions
Kojima eta al., 2020 [24]	3 studies (2 cross-sectional, and one prospective)	Joanna Briggs Institute Critical Appraisal Checklist (for Analytical Cross-Sectional Studies) and Newcastle-Ottawa scale (NOS, for cohort studies). All studies were considered to have adequate methodological quality.	Qualitative analysis	Pet ownership may be associated with a lower risk of frailty. The two cross sectional studies showed that those taking care of pets had a lower risk of being frail compared to those who were not. The prospective study showed that past dog owners had lower odds of developing frailty compared to never-dog owners but no statistical significance was reached for current pet owners. There were no significant differences in incident frailty risks in current and past cat owners compared to those in never cat owners.
Maurice et al., 2022 [25]	6 studies (5 cross-sectional studies and 1 before‐after with control group)	Newcastle Ottawa Scale (for observational epidemiological studies) – mean score: 6.8 out of 9; and Effective Practice and Organization of Care scale (EPOC, for the only clinical trials) – score: 2 out of 8.	Qualitative analysis	The studies on the effects of dog ownership on mental health reported mixed results: one study found a negative association with depression, anxiety, or loneliness; one study found a positive association with the feeling of happiness; 2 studies did not find a significant association with depression after adjusting for sociodemographic factors; one study found a positive association with an increase in the frequency of seeking outpatient mental care; one study found a positive association with symptoms of depression. 2 studies found a positive association with light-to-moderate physical activity.
Orr et a. 2023 [26]	34 studies (16 qualitative and 18 randomized trials)	Wallace criteria (for qualitative studies) and (Cochrane Risk of Bias Tool for quantitative studies) – most randomized trials were rated as low quality.	Thematic synthesis and random effect meta-analysis	Qualitative evidence synthesis identified 5 themes related to resident wellbeing: animals as ‘living beings’, reminiscence and storytelling, caring (as ‘doing’ and ‘feeling’), respite (from loneliness, institutionalization, and illness), and sensory engagement. A sixth theme related to staff perceptions and wellbeing, and a seventh to animal health and wellbeing. Maintaining identity was identified as an overarching theme. Quantitative studies showed limited evidence of a positive effect of animal interaction on outcomes of loneliness, anxiety, and depression, but the majority of randomized trials showed no significant effect. Meta-analyses conducted for the outcomes of depression, anxiety, agitation, and quality of life showed a significant reduction in anxiety. SMD − 0.36 (−0.68 to −0.04, *p* = 0.03; I2 = 5%).
Reniers et al., 2022 [27]	15 qualitative studies	Mixed-Methods Assessment Tool (MMAT) was used to assess the quality of the included studies	Interactive-inductive thematic analyses in ATLAS.it	28 themes grouped into 7 categories: (a) relational aspects (attachment, unconditional love, and interdependence), (b) reflection and meaning (attribution of feelings, saved memories, sense of achievement from taking care of pets, and meaning of life), (c) emotional aspects (responsiveness to feelings, emotional support, pleasure, and grief), (d) aspects of caregiving (need to focus on something other than themselves, responsibility of making plans for the pets in case of death or being away from home, worries for pets’ health, pet-related complaints like noise complaints, increased risk of falls, pet-related expenses), (e) physical health (exercise, daily routine, distraction from physical pain, relaxation and medical detection), (f) social aspects (reduced feelings of loneliness, and active or passive social facilitation), (g) bidirectional behavior (responsiveness to each other’s behavior, mirroring, and proximity). Overall, the older adults indicated that they had a strong bond with their pets, and that they believed their pets had a positive influence on their social, mental, and physical wellbeing. Some participants also highlighted negative aspects of pet ownership categorized as “aspects of caregiving”.
Scoresby et al., 2021 [28]	15 quantitative studies (11 cross-sectional, 3 prospective, 1 quasi experimental)	Article Quality Index based on two previous systematic reviews (0–9 points). Mean score for reviews focusing on older adults = 6,2	Qualitative analysis	15 studies compared mental health outcomes in pet owners to non-pet owners: 2 found a negative impact, 7 found mixed results, 5 found no impact. 1 study examined mental health outcomes in relation to the pet owner’s attachment bond with their pet and found no impact.

### Methodological quality of the included reviews

The methodological quality of the included systematic reviews was assessed as follows: critically low for three reviews [5, 24, 28], low for two reviews [10, 26], moderate for one review [25], high for one review [27].

### Findings of quantitative research

Primary research studies were grouped into two main categories: AASP, which were conducted in care living facilities, and pet ownership, which focused on community-dwelling older adults living at home with their pets and includes mostly studies with a cross-sectional design.

Five different types of exposures were identified: (a) dog-assisted support programs (interventions conducted with dogs alone or with dogs plus other animals); (b) AASP (based on animals other than dogs); (c) dog ownership (studies conducted on older adults owning a dog with or without other pets); (d) cat ownership (studies conducted on older adults owning a cat but not a dog); (e) pet ownership (studies conducted on pet owners without any specification of the type of pet owned).

Table 3 presents the outcomes of quantitative studies for the different exposures. A total of nine main outcomes were identified for both emotional/mental health (depression, apathy, anxiety, dementia/cognitive function, agitation, daily functionality, quality of life, loneliness, social interaction) and physical health (general physical health, cardiovascular mortality, balance/falls, frailty, daily circadian cortisol cycle, nutrition, blood pressure, heart rate variability, physical activity).

Dog-assisted support programs displayed positive effects on loneliness (in all four studies assessing this outcome) [67–70] and social interaction (in six studies out of nine) [37, 59, 71–77]. Positive effects of dog-assisted support programs were also found on blood pressure, although only two studies assessing this outcome were found [98, 101]. Two studies evaluating the effects of aquariums placed in the dining room of a nursing home reported positive effects on nutrition and body weight [82, 104].

Studies on pet ownership reported that dog owners appeared to engage in higher levels of physical activity in 10 studies out of 12 (one study found mixed effects and one found no effects) [85–88, 95, 105, 106, 111, 114–117] and to be less fragile in two studies [85, 109].

Most of the analyzed outcomes showed mixed results. In particular, mixed results emerged when considering dog-assisted support programs and depression [29–48], apathy [33, 36, 49], anxiety [29, 33, 34, 40, 50], dementia/cognitive function [31, 35, 36, 40, 45, 48–56], agitation [30, 32, 36, 40, 53, 57–59], daily functionality [36, 48, 50, 52, 60, 61], quality of life [32, 33, 46, 62–66], balance/falls [52, 64], and physical activity [36, 103]. AASP showed mixed results for depression [78–81], anxiety [78–80], dementia/cognitive function [78, 79, 81–83] and social interaction [41, 80, 84]. Results of studies on dog ownership were mixed concerning the association with depression [85–92], dementia/cognitive function [88, 93], daily functionality [87, 94], loneliness [89, 90, 95, 96], social interaction [85, 89, 90, 95, 96], general physical health [85, 86, 92, 95, 96, 105–107] and blood pressure [110–112]. No association appeared between dog ownership and anxiety in the two studies assessing this outcome [86, 89]. Research on cat ownership showed mixed results for depression [85, 87–93, 97], dementia/cognitive function [88, 93, 97], daily functionality [87, 94], social interaction [85, 89, 90, 95], general physical health [85, 92, 95, 107], blood pressure [110, 112], and physical activity [85, 87, 88, 95, 116, 117]; no association occurred between cat ownership and loneliness [89, 90, 95, 97] and frailty [85, 109]. Finally, pet ownership was not associated with general physical health [14, 16], and mixed results emerged with regard to depression [14, 93, 98, 99].

**Table 3:** Summary of evidence for quantitative research synthesis: number of primary studies with positive, null, negative or mixed associations grouped for different outcomes and type of exposure. Outcomes with more than one study are presented with a visual indicator (green: beneficial association; yellow: no association)

**Emotional and mental health**									
	**Depression**	**Apathy**	**Anxiety**	**Dementia/cognitive function**	**Agitation**	**Daily functionality (i.e. ADL, self-care)**	**Quality of life**	**Loneliness**	**Social interaction**
Dog-assisted support programs	+: 8 [29–36]null: 9 [37–45]+/-: 2 [46, 47]-: 1 [48]	+: 2 [33, 49]null: 1 [36]	+: 3 [33, 40, 50]null: 2 [29, 34]	+: 4 [31, 35, 48, 51]null: 8 [36, 40, 45, 49, 52–55]+/-: 2 [50, 56]	+: 3 [30, 57, 58] null: 4 [32, 36, 40, 59]-: 1 [53]	+: 1 [48]null: 5 [36, 50, 52, 60, 61]	+: 4 [33, 46, 62, 63]+/-: 1 [32]null: 3 [64–66]	+: 4 [67–70]	+: 6 [59, 71–75]null: 3 [37, 76, 77]
AASP	+: 2 [78, 79]null: 2 [80, 81]	//	+: 1 [78] null: 2 [79, 80]	+: 3 [79, 82, 83]null: 2 [78, 81]	//	+: 1 [78]	+: 1 [78]	//	+: 2 [41, 84]+/-: 1 [80]
Dog ownership	+: 2 [85, 86]null: 4 [87–90]-: 2 [91, 92]	//	null: 2 [86, 89]	+: 1 [93]null: 1 [88]	//	+: 1 [94]null: 1[87]	//	+: 1 [90]null: 3 [89, 95, 96]	+: 2 [85, 90]null: 2 [89, 95]+/-: 1 [96]
Cat ownership	null: 7 [85, 87–90, 93, 97]-: 2 [91, 92]	//	null: 1 [89]	+: 1 [93]null: 2 [88, 97]	//	+: 1 [94]null: 1 [87]	//	null: 4 [89, 90, 95, 97]	+: 1 [85] null: 3 [89, 90, 95]
Pet ownership	+: 2 [98, 99]-: 1 [14] null 1: [93]	//	//	null: 1 [100]	+: 1 [100]	//	//	-: 1 [87]	//
**Physical health**									
	**General physical health**	**Cardiovascular mortality**	**Balance/falls**	**Frailty**	**Daily circadian cortisol cycle**	**Nutrition and body weight**	**Blood pressure**	**Heart rate variability**	**Physical activity**
Dog-assisted support programs	+/-: 1 [46]	//	+: 1 [64]-: 1 [52]	//	-: 1 [38]	-: 1 [52]	+: 2 [98, 101]	+: 1 [102]	+: 1 [103]-: 1 [36]
AASP	+: 1[79]	//	//	//	-: 1 [83]	+: 2 [82, 104]	+: 1 [81]	//	//
Dog ownership	+: 3 [85, 86, 105]null: 3 [95, 106, 107]+/-: 1 [96]-: 1 [92]	+: 1 [108]	//	+: 2 [85, 109]	//	//	+: 1 [110]null: 2 [111, 112]	+: 1 [113]	+: 10 [85–87, 95, 106, 111, 114–117]+/-: 1 [105]null: 1 [88]
Cat ownership	null: 3 [85, 95, 107]-: 1 [92]	+: 1 [108]	//	null: 2 [85, 109]	//	//	+/-: 1 [110]null: 1[112]	+: 1 [113]	null: 3 [85, 87, 88]-: 3 [95, 116, 117]
Pet ownership	null: 2 [14, 16]	+: 1 [118]		+: 1 [119]	//	//	//	+: 1 [120]	-: 1 [112]

**Note:** + = positive effect or beneficial association; - = negative effect or unfavorable association; +/- = mixed effect or association; null = no statistically significant effect or association

### Findings of qualitative research

Findings of qualitative research are presented in Table 4. Six main themes (reminiscing and meaning [121–133], sensory engagement [121, 123–125, 127–129, 132–137], emotional aspects [121–128, 130–138], relational aspects [122, 123, 126–132], organizational aspects and staff’s perspective [121, 123, 126, 129, 133, 134, 136], and animal wellbeing [123, 128, 130, 133–136]) were found for AASP conducted in living facilities and seven for pet ownership (relational aspects [11, 131, 139–152], reminiscing and meaning [11, 131, 139–144, 147–152], emotional aspects [11, 131, 140–152], aspects of caregiving [11, 140–144, 146–152], physical health [11, 140–142, 144–146, 148–152], social aspects [11, 139, 141–144, 146–152], bidirectional behavior [11, 140–142, 144, 147–152]). Some of the identified themes were shared in both the exposures, although they were expressed in different shades, and others were only typical of the specific context. 

“Reminiscing and meaning” was a theme present in both AASP [121–133] and pet ownership [11, 139–144, 147–152] according to which animals enabled the older adults to engage in the recollection of memories from the past associated with family members or events. The recollection of past experiences was found to be important, particularly for older adults living in care facilities, since it helped their expression, the connection with past self, and maintaining a sense of self-identity [121–132].

“Emotional aspects” was another theme reported by both residents of the facilities that participated in AASP [121–128, 130–138] and pet owners [11, 140–152]; according to them, animals offered emotional support, reduced feelings of loneliness, and gave a sense of engagement and involvement in meaningful activities. Regarding this theme, pet-ownership was also related to periods of grief, particularly during a pet’s illness or after a pet’s death [140, 144, 147, 149, 151].

Older adults living in facilities benefitted from the “sensory engagement” derived from the human-animal interaction that could provide pleasure, comfort, calmness, and responded to the need for physical contact [121, 123–125, 127–129, 132–137]. Also, AASP offered opportunities for reciprocal interactions between staff members, older adults, relatives and volunteers; pets could act as substitutes for human relationships in interactions that are perceived as non-judgmental [122, 123, 126–132]. These aspects were recollected in the “relational aspects” theme.

AASP conducted in care living facilities had two other specific themes: “organizational aspects and staff’s perspective” [121, 123, 126, 129, 133, 134, 136], and “animal wellbeing” [123, 128, 130, 134, 135]. Concerning the first theme, some of the staff members reported that the presence of animals created a positive work environment and that, thanks to the animals, they experienced a more person-centered approach, cared for, and reached the residents on a cognitive level rather than just responding to physical care needs [123, 131, 136]. On the other hand, some staff members felt that the presence of animals was troublesome in the routines of the living facilities and could distract them from caring for the residents due to the additional work required [121, 134, 136]. In addition, organizational problems were identified related to the risks of reduced hygiene and safety hazards for residents who fall over animals or are allergic to animals [121, 134].

Finally, the use of animals in AASP raised the problem of meeting ethical standards to guarantee the animal wellbeing and mutually beneficial interactions for both the animals and the older adults [123, 128, 130, 133–136].

Among the pet owners, the “relational aspects” theme was more focused on the human-animal bond, with the feeling of a bidirectional relation and interdependence between the owner and the pet [11, 139–152]; the promotion of interhuman interaction due to the animals (i.e. meeting people during dog walking or joining virtual communities of pet lovers in the social networks) was included in the “social aspects” theme [11, 139, 141–144, 146–152].

Pet ownership was also related to “aspects of caregiving” that offered to older adults the opportunity to focus on a living being other than themselves and provided a sense of safety (for example, from the barking of dogs) [11, 140–144, 146–152]. This category also included some negative aspects such as worries related to the care of the pet if they had to stay away from home for a prolonged period (i.e. some older adults reported that they considered postponing a hospitalization if they had no trustworthy person to care for the pet), pet-related complaints (i.e. noise complaints), worries for the pet’s health, and pet related expenses [11, 140–142, 146, 148, 150–152]. 

Concerning the “physical health” theme, pet owners perceived that pets were beneficial for their health by spurring them on to additional exercise, creating a daily routine, offering a distraction from self-physical pain and through medical detection (a phenomenon whereby some pets are perceived to detect upcoming medical events) [11, 140–142, 144–146, 148–152]. 

Finally, in the “bidirectional behavior” theme, pets and owners appeared to be responsive towards each other’s behavior (i.e. owners liked to pet and hug their animals and pets actively sought to be touched by their owners) [11, 140–142, 144, 147–152]. Pets seemed to help some older adults to be more aware of their surroundings [11, 141, 142, 144, 147–152], and some owners reported seeing their personality traits reflected in their pets [11, 142, 144, 147–149, 151].

**Table 4:** Findings from qualitative synthesis

Exposure	Synthesized finding	Details
Animal-assisted support programs	Reminiscing and meaning [121–133]: - memories - storytelling - expression	Animals recall happy or sad memories of family, past pets, former occupations, and prompt the expression of personal recollections, memories, and storytelling with other people. Recollection of past events and experiences helps connect to past self and maintain a sense of self-identity
	Sensory engagement [121, 123–125, 127–129, 132–137]: - physical touch and comfort - expressing oneself through the senses	Human-animal interaction can provide pleasure and joy, comfort, calmness, and respond to the need for physical contact. The presence of animals offers a sensory experience, primarily by tactile and visual stimulation.
	Emotional aspects [121–128, 130–138]: - emotional support and respite - responsiveness - sense of engagement	Pets provide emotional support, reduce feelings of loneliness, offer relief from physical pain and anxiety, and reduce stress. Caring for pets creates a sense of engagement and promotes the involvement in meaningful activities
	Relational aspects [122, 123, 126–132]: - stimulus for human interactions - supplement missing interactions - human-animal bonding	Animals offer opportunities for reciprocal interactions between staff, older adults, relatives, and volunteers. Animals are described as transitional objects to supplement missing interaction by “filling a void”. Pets could act as substitutes for human relationships; interactions with animals are perceived as non-judgmental.
	Organizational aspects and staff’s perspective [121, 123, 126, 129, 133, 134, 136]: - enhanced workplace - readiness for visits to take place - controlling any risk factors - hygiene - increased workload	The presence of animals can create a positive environment in nursing homes. Staff members experienced a more person-centered approach to care and reached the residents on a cognitive level rather than just responding to physical care needs. Some staff members can feel that the presence of animals is disruptive to the routines of the care home and could distract them from caring for the residents due to the additional work required. Other issues for staff are risks of reduced hygiene and safety hazards from residents falling over animals or being allergic to animals.
	Animal wellbeing [123, 128, 130, 133–136]: - fulfilling role - enjoyment - potential for distress	Ethical standards for the use of animals in interventions must be met. Reciprocity and mutually beneficial interactions for both the animals and the older adults must be encouraged.
Pet ownership	Relational aspects [11, 139–152]: - attachment - unconditional love - interdependence	Feeling of a bidirectional bond; pets are non-judgmental and always available; pets rely on the owners for care and the owners rely on the support and affection of their pets.
	Reminiscing and meaning [11, 139–144, 147–149, 149, 149–152]: - attribution to feelings - memories - meaning of life	Pets are perceived to have human-like feelings; owners save important memories with pets, or pets are associated in memories with deceased family members.
	Emotional aspects [11, 140–149, 149–152]: - responsiveness to feelings - emotional support - pleasure - grief	Pets perceive the feelings of their owners and offer emotional support; owners undertake fun activities with their pets; pet-ownership is related to periods of grief particularly after a pet’s death.
	Aspects of caregiving [11, 140–144, 146–152]: - need of care giving - responsibility - sense of safety - expenses - worries	Caregiving provides an opportunity to focus on something other than self, but some older adults need help from other people to care for their pets; the barking of dogs provides a sense of safety; owners feel responsible for their pets and worry if they have to stay away for too long (i.e. they might postpone hospitalizations if they have no trustworthy person to care for their pets); other worries include pet death, noise complaints, increased risk of falls due to the pet and pet-related expenses.
	Physical health [11, 140–142, 144–146, 148–152]: - exercise - daily routine - distraction from physical pain - relaxation - medical detection	Pets are perceived as beneficial for owners’ physical health through additional exercise. They impose a daily routine, and the need to focus on the pet can distract attention from physical pain and help to relax; some pets are perceived to detect and warn their owners about upcoming medical events
	Social aspects [11, 139, 141–144, 146–152]: - feelings of loneliness - passive social facilitation - active social facilitation	Pets reduce feelings of loneliness, offer company, and connect the owners to other people (i.e. meeting people during dog walking or joining virtual communities of pet lovers in social networks).
	Bidirectional behavior [11, 140–142, 144, 147–152]: - responsiveness to behavior - physical contact - proximity - mirroring	During daily routines and interactions, pets and owners are responsive to each other’s behavior. Pets might help some older adults to be more aware of their surroundings. Owners pet and hug their pets and like to be close to them. The pets actively seek to be touched by their owners. Some older adults see their own personality traits reflected in their pets.

## Discussion

Overall, the studies included in this umbrella review reported mixed findings regarding the effects of AASP and pet ownership on various aspects of health and well-being in older adults. While several primary studies highlighted potential benefits, many others showed inconsistent or inconclusive results across different types of programs and pet ownership. These mixed findings underscore the complexity of the relationship between human–animal interaction and health in older populations and suggest the need for cautious interpretation and further high-quality research.

In interpreting the mixed findings, it is crucial to consider the broad heterogeneity in the types of exposures and interactions evaluated across the included studies. Differences in species (e.g., dogs, cats, or other animals), the nature of the interaction (e.g., structured AASP vs. unstructured pet ownership), and the context (e.g., individual vs. group settings) likely contribute to the variability in outcomes. Moreover, the personal characteristics and life contexts of older adults may modulate how these interactions are experienced and perceived. This diversity suggests that a “one-size-fits-all” approach is not applicable, and that future research and interventions should carefully account for the type and quality of human–animal interactions when targeting specific health outcomes.

Also, the variability in study findings may, in part, be attributed to the limited sample sizes of many primary studies included in the reviews. Small samples can reduce statistical power and increase the likelihood of inconsistent or inconclusive results, particularly for complex and multifactorial outcomes such as mental health or cognitive function.

This umbrella review found positive impacts of dog-assisted support programs mainly on loneliness, social interaction, and blood pressure in older adults. Concerning physical health, dog owners appeared to have higher levels of physical activity and to be less frail than the controls. Generally, dogs’ companionship has been shown to be more beneficial compared to that of other animals, apart from the association with the nutrition of older adults living in facilities which benefited from the presence of aquariums placed in the dining room [82, 104], but not from the presence of cats or dogs [52].

Studies investigating the associations of pet visits on social interactions elucidated that the pet triggered physical touch, eye contact, and verbal communication among older adults living in care facilities [71–73]. The positive effects of AASP on social interactions can be explained through a complex interplay of various factors. Of central importance is the communication between humans and animals, which happens largely via the reception and interpretation of visual, tactile, auditory, and olfactory stimuli and signals [73]. One study comparing the responses triggered by a real dog, a robot seal, and a toy cat, found higher levels of social interaction in response to the real dog and the robot seal, compared to the toy cat, even though the interest in the robot seal decreased during the intervention period [71]. This finding suggests that the ability of the animal (or the animated object) to interact and give feedback affects the response of the participants; the decreased interaction over time with the robot seal suggests that the object did not maintain the attention of the participants at the same level as the dog, probably due to the less spontaneous responses and limited behavioral repertoire [71]. Additionally, AASP offers biographical stimuli, building on emotionally important content from long-term memory: animals frequently elicit positive memories in older people, for example, of previous pets or past life events they shared with their pets [26]. This aspect is well documented in the results of qualitative research that emphasizes the role of visiting pets in promoting reminiscence and recollection of past experiences [10, 26]. Reminiscence also served older adults as a way of connecting to past selves and helped to maintain a sense of self and identity [26]. Moreover, animals were able to bridge communication between humans since they served as a reason for and a topic of communication, and they enhance interpersonal contact as so-called “social catalysts” [73].

In addition to increasing socialization, AASP conducted in long-term care facilities showed that dogs can have a positive effect on loneliness [67–70]. The exact mechanisms by which AASP result in decreased loneliness are unclear; previous studies attributed it to the increase of human social interaction facilitated by the dog [154] or to the attachment to the pet that participants develop through the interventions [155]. Both of these theories were rejected by the works of Banks et al., which showed that the major benefit of AASP was found when it was conducted in an individual setting instead of in a group setting [68] and that attachment did not correlate with the change in loneliness, suggesting that it does not account for the dog’s ability to reduce loneliness [69]. Interestingly, an association between pretest and posttest loneliness scores and between pretest and delta loneliness was found, indicating that those who were most lonely improved the most [68, 69]. This association was also found for the control group, indicating that the loneliest who did not receive AASP became increasingly lonely [68, 69]. Results from the qualitative research showed that animals were often described as “effective transitional objects to supplement missing interaction by either filling a void or supplementing other human interactions and bonds” [10]. Again, older adults living in long-term care facilities reported that pets seemed to counteract their loneliness and help them make friends with other residents and staff [26]: a shared interest in animals enabled some residents to build relationships, animals could be a “talking point” that staff and residents “enjoyed together”, and the presence of animals could lead to enhanced interactions between residents themselves and a greater interest in each other [26].

Although only two studies evaluated the effects of AASP on blood pressure, their findings appear to support the beneficial effects of dog presence: both studies found that the participants’ blood pressure was lower when the dog was present inside the room [98, 101], suggesting that the pet might provide a viable means of decreasing cardiovascular reactivity and stress responses in older adults [101]. These findings regarding the effects of pet ownership on blood pressure are consistent with previous research suggesting a potential buffering role of pets in stress-related physiological responses. For example, one study found that pet owners exhibited significantly lower resting heart rate and blood pressure, smaller increases in response to stressors (e.g., mental arithmetic and cold pressor tasks), and faster recovery compared to non-pet owners [9]. Notably, the lowest cardiovascular reactivity and the quickest recovery were observed when pets were physically present during the tasks [9]. These findings support the idea that the presence of a pet may modulate physiological stress responses.

Most of the studies (ten out of twelve) on pet ownership and physical activity showed that dog owners had higher levels of physical activity than non-dog owners, including owners of other types of pets. This result is attributed to dog-walking; in fact, dog owners were more likely to walk more frequently, for longer periods of time, and to participate in increased levels of leisure physical activity than non-dog owners [156, 157]. Related to this, a study found that due to the characteristics of the walking performed by dog owners, they did not meet physical activity guidelines (≥ 7.5 MET-hr/wk), possibly related to the low walking intensity performed [105]. Indeed, dog owners were more likely to engage in casual strolling/walking and less likely to walk fairly fast or very fast than non-dog owners [105, 158, 159]. Two studies found that dog owners also appeared to have lower levels of frailty [85, 109]. Frailty is a state in which cumulative aging-related deficits decrease reserve across multiple physiological systems and make individuals more vulnerable to internal and external stressors, increasing risks of adverse health outcomes [160, 161]. Although the mechanism for frailty development remains unclear, social frailty (i.e., going out less frequently, rarely visiting friends, feeling less like helping friends or family, living alone, and not talking to anyone all day) may precede and lead to the development of physical frailty [162]. The experience of dog ownership might reduce the risk of incident frailty in later life through a physically and socially active lifestyle [85].

Many studies focused on the effects of animal companionship on depression and anxiety; although some of them found a negative association indicating that pets might have a beneficial effect on older adults, many others failed to highlight this association. In this regard, a study on dog ownership found no significant group differences for depression or anxiety among the dog owners compared with non-dog owners, but when the frequency of dog presence was considered, a strong negative association with depression and a moderate negative association with anxiety and loneliness were found [89]. Analyzing the characteristics of the human-animal relationship in even more detail, another study reported that pet engagement, but not pet availability, manifested a significant negative effect on depression [99]. In other words, the mere presence of a pet would not be enough to prevent depression; instead, engagement with the pet is required to bolster prevention [163]. The predictive effect of pet engagement on low depression illustrates the interaction principle in ecological theory, which states that interaction or engagement with the environment or its members is necessary to acquire environmental resources [164]. Nonetheless, resources provided by the pet unconditionally tended to be limited in their usefulness, because they hinge on the reaction of the older adult [163, 165]. For instance, if one did not feel competent in or proud of engaging with a pet, one would not benefit from the engagement.

While quantitative studies did not show negative associations between pet exposure and health outcomes, apart from individual studies or very mixed results, qualitative research highlighted some negative aspects of pet ownership. In particular, older adults reported concerns regarding pets’ health, pet-related expenses, complaints from other people due to the pet (i.e., for pets’ noise), and worries related to the responsibility of caring for the animal [27]. However, most of the pet owners’ perceptions about their pets were positive. According to older adults’ own experiences, pets play an important and beneficial role in their lives: older adults reported additional social connections, emotional support, and physical activities resulting from pet ownership [27]. Both the positive and negative experiences highlight the importance of considering pets in the care system of older adults.

### Strengths and limitations

Based on our findings, this review constitutes the most all-encompassing and recent investigation into the impact of animals on the health and wellbeing of older adults. There were no restrictions imposed on the population regarding various environments (such as long-term care facilities or community settings) or forms of human-animal interaction (including pet ownership or AASP), which enabled us to gather and analyze findings from all existing research on this subject. Furthermore, incorporating qualitative research allowed for a more profound understanding of how older individuals perceive their relationships with animals, highlighting the importance of documenting the variety, richness, and commonalities in their experiences with animal companionship. A significant limitation of the umbrella review arises from the extensive variety of measured variables and the inconsistencies in study designs, making it difficult to draw comparisons between the results of different studies.

The discrepancies observed between qualitative and quantitative findings may, in part, be due to limitations in the outcome measures used in quantitative studies. Specifically, many standardized instruments may lack suitability, that is, they may not align well with the kinds of benefits older adults experience from human-animal interactions, such as emotional comfort, sense of engagement, or attachment. Additionally, some tools may lack responsiveness or sensitivity to detect subtle but meaningful changes over time. In contrast, qualitative studies often captured rich descriptions of personal experiences and perceived impacts that may not be fully reflected in quantitative scores.

It is possible that the limited sample sizes in certain studies were insufficient to identify significant variations. It is also conceivable that the variation can be attributed to the broad and varied types of interactions between humans and animals that have been examined and evaluated in different research studies. The species of animal involved, the dynamics of the engagement (whether solitary or group-based, AASP versus traditional pet ownership), along with the characteristics and backgrounds of the older adults, inevitably result in a diverse range of experiences and effects that are challenging to quantify and unlikely to be consistent.

## Conclusions

In conclusion, quantitative studies showed that exposure to pets, and in particular dogs, appeared to bring beneficial impacts on social interaction and loneliness in older adults living in care facilities. Dog owners benefited from pets’ companionship, particularly regarding the higher levels of physical activity due to dog walking. Qualitative studies highlighted a variety of beneficial associations derived from pet ownership and AASP, with some negative aspects related to the worries, responsibilities and expenses of pet ownership. These outcomes can serve as a conceptual framework to develop guidelines, tools, and interventions involving companion animals, preferably in collaboration with stakeholders such as managers and organizational staff of nursing homes and home care programs, older adults and family caregivers.

## Data Availability

No datasets were generated or analysed during the current study.

## Appendix 1

**Table 5:** PRISMA 2020 checklist

Section and Topic	Item #	Checklist item	Location where item is reported
TITLE			
Title	1	Identify the report as a systematic review.	Page 1
ABSTRACT			
Abstract	2	See the PRISMA 2020 for Abstracts checklist.	Page 2
INTRODUCTION			
Rationale	3	Describe the rationale for the review in the context of existing knowledge.	Pages 4-6
Objectives	4	Provide an explicit statement of the objective(s) or question(s) the review addresses.	Page 6
METHODS			
Eligibility criteria	5	Specify the inclusion and exclusion criteria for the review and how studies were grouped for the syntheses.	Pages 8, 9
Information sources	6	Specify all databases, registers, websites, organisations, reference lists and other sources searched or consulted to identify studies. Specify the date when each source was last searched or consulted.	Page 7
Search strategy	7	Present the full search strategies for all databases, registers and websites, including any filters and limits used.	Pages 38, 39
Selection process	8	Specify the methods used to decide whether a study met the inclusion criteria of the review, including how many reviewers screened each record and each report retrieved, whether they worked independently, and if applicable, details of automation tools used in the process.	Page 8
Data collection process	9	Specify the methods used to collect data from reports, including how many reviewers collected data from each report, whether they worked independently, any processes for obtaining or confirming data from study investigators, and if applicable, details of automation tools used in the process.	Page 9
Data items	10a	List and define all outcomes for which data were sought. Specify whether all results that were compatible with each outcome domain in each study were sought (e.g. for all measures, time points, analyses), and if not, the methods used to decide which results to collect.	Page 9
	10b	List and define all other variables for which data were sought (e.g. participant and intervention characteristics, funding sources). Describe any assumptions made about any missing or unclear information.	Page 9
Study risk of bias assessment	11	Specify the methods used to assess risk of bias in the included studies, including details of the tool(s) used, how many reviewers assessed each study and whether they worked independently, and if applicable, details of automation tools used in the process.	Pages 10
Effect measures	12	Specify for each outcome the effect measure(s) (e.g. risk ratio, mean difference) used in the synthesis or presentation of results.	Page 9
Synthesis methods	13a	Describe the processes used to decide which studies were eligible for each synthesis (e.g. tabulating the study intervention characteristics and comparing against the planned groups for each synthesis (item #5)).	Page 9
	13b	Describe any methods required to prepare the data for presentation or synthesis, such as handling of missing summary statistics, or data conversions.	Not applicable
	13c	Describe any methods used to tabulate or visually display results of individual studies and syntheses.	Page 9
	13d	Describe any methods used to synthesize results and provide a rationale for the choice(s). If meta-analysis was performed, describe the model(s), method(s) to identify the presence and extent of statistical heterogeneity, and software package(s) used.	Page 9
	13e	Describe any methods used to explore possible causes of heterogeneity among study results (e.g. subgroup analysis, meta-regression).	Not applicable
	13f	Describe any sensitivity analyses conducted to assess robustness of the synthesized results.	Not applicable
Reporting bias assessment	14	Describe any methods used to assess risk of bias due to missing results in a synthesis (arising from reporting biases).	Not applicable
Certainty assessment	15	Describe any methods used to assess certainty (or confidence) in the body of evidence for an outcome.	Not applicable
RESULTS			
Study selection	16a	Describe the results of the search and selection process, from the number of records identified in the search to the number of studies included in the review, ideally using a flow diagram.	Page 11
	16b	Cite studies that might appear to meet the inclusion criteria, but which were excluded, and explain why they were excluded.	Page 11
Study characteristics	17	Cite each included study and present its characteristics.	Pages 11, 26-34
Risk of bias in studies	18	Present assessments of risk of bias for each included study.	Pages 12, 40
Results of individual studies	19	For all outcomes, present, for each study: (a) summary statistics for each group (where appropriate) and (b) an effect estimate and its precision (e.g. confidence/credible interval), ideally using structured tables or plots.	Not applicable
Results of syntheses	20a	For each synthesis, briefly summarise the characteristics and risk of bias among contributing studies.	Pages 26-34, 40
	20b	Present results of all statistical syntheses conducted. If meta-analysis was done, present for each the summary estimate and its precision (e.g. confidence/credible interval) and measures of statistical heterogeneity. If comparing groups, describe the direction of the effect.	Pages 12-16, 31-34
	20c	Present results of all investigations of possible causes of heterogeneity among study results.	Not applicable
	20d	Present results of all sensitivity analyses conducted to assess the robustness of the synthesized results.	Not applicable
Reporting biases	21	Present assessments of risk of bias due to missing results (arising from reporting biases) for each synthesis assessed.	Not applicable
Certainty of evidence	22	Present assessments of certainty (or confidence) in the body of evidence for each outcome assessed.	Not applicable
DISCUSSION			
Discussion	23a	Provide a general interpretation of the results in the context of other evidence.	Pages 17-23
	23b	Discuss any limitations of the evidence included in the review.	Pages 22, 23
	23c	Discuss any limitations of the review processes used.	Pages 22, 23
	23d	Discuss implications of the results for practice, policy, and future research.	Page 23
OTHER INFORMATION			
Registration and protocol	24a	Provide registration information for the review, including register name and registration number, or state that the review was not registered.	Page 7
	24b	Indicate where the review protocol can be accessed, or state that a protocol was not prepared.	Page 7
	24c	Describe and explain any amendments to information provided at registration or in the protocol.	Not applicable
Support	25	Describe sources of financial or non-financial support for the review, and the role of the funders or sponsors in the review.	Page 24
Competing interests	26	Declare any competing interests of review authors.	Page 24
Availability of data, code and other materials	27	Report which of the following are publicly available and where they can be found: template data collection forms; data extracted from included studies; data used for all analyses; analytic code; any other materials used in the review.	Page 24

## Appendix 2. Research search strings

### PubMed search string

(("health"[MeSH Terms] OR "health"[Title/Abstract] OR "physical health"[Title/Abstract] OR "mental health"[Title/Abstract] OR"emotional health"[Title/Abstract] OR "Quality of Life"[MeSH Terms] OR "Quality of Life"[Title/Abstract] OR"well-being"[Title/Abstract] OR "wellbeing"[Title/Abstract] OR "wellness"[Title/Abstract]) AND ("old people"[Title/Abstract] OR "old adult*"[Title/Abstract] OR "old age"[Title/Abstract] OR "older people"[Title/Abstract] OR "older adult*"[Title/Abstract] OR "older age"[Title/Abstract] OR"geriatric*"[Title/Abstract] OR "elder*"[Title/Abstract] OR"senior*"[Title/Abstract] OR "older person*"[Title/Abstract] OR "old person*"[Title/Abstract] OR"aging adult*"[Title/Abstract] OR "aging person*"[Title/Abstract] OR "Aged"[MeSH Terms]) AND (((("Pets"[MeSH Terms] OR "pet"[Title/Abstract] OR "Pets"[Title/Abstract] OR "dogs"[Title/Abstract] OR "dog"[Title/Abstract] OR"cat"[Title/Abstract] OR "cats"[Title/Abstract] OR"pet ownership"[Title/Abstract] OR "companion animal*"[Title/Abstract] OR "domestic animal*"[Title/Abstract] OR "emotional support animal*"[Title/Abstract] OR "comfort animal*"[Title/Abstract]) NOT "therapy animals"[MeSH Terms]) NOT"service animals"[MeSH Terms]) NOT "animal assisted therapy"[MeSH Terms]) AND ("meta analysis"[Publication Type] OR "systematic review"[Filter])) AND (meta-analysis[Filter] OR review[Filter] OR systematicreview[Filter])

### PsycINFO search string, EBSCO database

AB (("health" OR"physical health" OR "mental health" OR "emotional health" OR "quality of life” OR “wellbeing” OR “wellness” OR“well-being”)) AND AB ((“old people” OR “old adult*” OR “old age” OR “older people” OR “older adult*” OR “older age” OR "geriatric*" OR"elder*" OR "senior*" OR older person* OR old person* OR aging adult* OR aging person* OR "Aged")) AND AB ((“pets” OR “pet” OR "dogs" OR "dog" OR "cats" OR "cat" OR "pet ownership” OR "companion animal*" OR "comfort animal" NOT "therapy animals” NOT “service animals” NOT “animal assisted therapy”)). Narrow by Methodology: - meta analysis. Narrow by Methodology: - systematic review

### Scopus search string

(TITLE-ABS (health) OR TITLE-ABS (physical AND health) OR TITLE-ABS (mental AND health) OR TITLE-ABS (emotional AND health) OR TITLE-ABS (quality AND of AND life) OR TITLE-ABS (wellbeing) OR TITLE-ABS (wellness) TITLE-ABS (well-being)) AND (TITLE-ABS (old AND people) OR TITLE-ABS (old AND adult*) OR TITLE-ABS (old AND age) OR TITLE-ABS (older AND people) OR TITLE-ABS (older AND adult*) OR TITLE-ABS (older AND age) OR TITLE-ABS (geriatric*) OR TITLE-ABS (elder*) OR TITLE-ABS (senior*) OR TITLE-ABS (older AND person*) OR TITLE-ABS (old AND person*) OR TITLE-ABS (aging AND adult*) OR TITLE-ABS (aging AND person*) OR TITLE-ABS (ageing AND adult*) OR TITLE-ABS (ageing AND person*) OR TITLE-ABS (aged)) AND (TITLE-ABS (pets) OR TITLE-ABS (pet) OR TITLE-ABS (dog) OR TITLE-ABS (dogs) OR TITLE-ABS (cat) OR TITLE-ABS (cats) OR TITLE-ABS (pet AND ownership) OR TITLE-ABS (companion AND animal*) OR TITLE-ABS (comfort AND animal)) AND (LIMIT-TO (DOCTYPE,"re"))

### Web of Science search string

("Health" OR "physical health" OR "mental health" OR "emotional health" OR "quality of life" OR "wellbeing" OR"well-being" OR "wellness") AND ("old people" OR"old adult*" OR "old age" OR "older people" OR"older adult*" OR "older age" OR "geriatric*" OR"elder*" OR "senior*" OR older person* OR old person* OR aging adult* OR aging person* OR ageing person* OR "Aged") AND ("pets" OR "pet" OR "dog" OR "dogs" OR"cat" OR "cats" OR "pet ownership" OR"companion animal*" OR "comfort animal" NOT "therapy animals” NOT “service animals” NOT “animal assisted therapy”)

### Cochrane Library search string

("health" OR"physical health" OR "mental health" OR "emotional health" OR “wellbeing” OR “wellness” OR "quality of life") AND (“old people” OR old NEXT adult* OR “old age” OR “older people” OR older NEXT adult* OR “older age” OR geriatric* OR elder* OR senior* OR older NEXT person* OR old NEXT person* OR aging NEXT adult* OR aging NEXT person* OR"Aged") AND (“pets” OR “pet” OR "dogs" OR "dog" OR "cats" OR "cat" OR "pet ownership” OR companion NEXT animal* OR "comfort animal" NOT "therapy animals” NOT“service animals” NOT “animal assisted therapy”) in Title Abstract Keyword - (Word variations have been searched)

## Appendix 3

**Table 6:** Quality assessment according to the AMSTAR-2 scale

Main author, year	AMSTAR-2 items																
	1. Question and inclusion	2. Protocol*	3. Study design	4. Exhaustive search*	5. Study selection	6. Data extraction	7. Justification of excluded studies*	8. Details of included studies	9. Risk of bias*	10. Sources of founding	11. Statistical methods*	12. Risk of bias in meta-analysis	13. Risk of bias in individual studies*	14. Explanation of heterogeneity	15. Publication bias*	16. Conflict of interest	Global
Huges et al., 2020 [5]	Yes	No	No	Yes partial	Yes	No	Yes partial	Yes partial	No	No	No meta-analysis	No meta-analysis	No	No	No meta-analysis	Yes	Critically low
Jain et al., 2020 [10]	Yes	Yes partial	Yes	Yes	Yes	Yes	Yes partial	Yes partial	Yes partial	No	Yes	Yes	Yes	Yes	No	Yes	Low
Kojoma et al., 2020 [24]	Yes	Yes partial	Yes	Yes partial	No	No	No	Yes partial	No	No	No meta-analysis	No meta-analysis	Yes	Yes	No meta-analysis	Yes	Critically low
Maurice et al., 2022 [25]	Yes	Yes partial	Yes	Yes partial	Yes	No	Yes	Yes partial	Yes partial	No	No meta-analysis	No meta-analysis	Yes	Yes	Yes	Yes	Moderate
Orr et al., 2023 [26]	Yes	Yes partial	Yes	Yes partial	Yes	Yes	Yes partial	Yes partial	Yes partial	No	Yes	Yes	Yes	Yes	No	Yes	Low
Reniers et al., 2023 [27]	Yes	Yes partial	Yes	Yes partial	Yes	Yes	Yes partial	Yes partial	Yes partial	No	No meta-analysis	No meta-analysis	Yes	Yes	No meta-analysis	Yes	High
Scoresby et al. 2021 [28]	Yes	No	Yes	Yes partial	Yes	Yes	No	Yes partial	No	No	No meta-analysis	No meta-analysis	No	Yes	No meta-analysis	Yes	Critically low

*****AMSTAR-2 critical domains

### Rating overall confidence in the results of the review

 High

- *No or one non-critical weakness*: the systematic review provides an accurate and comprehensive summary of the results of the available studies that address the question of interest

Moderate

- *More than one non-critical weakness**: the systematic review has more than one weakness but no critical flaws. It may provide an accurate summary of the results of the available studies that were included in the review

Low

- *One critical flaw with or without non-critical weaknesses*: the review has a critical flaw and may not provide an accurate and comprehensive summary of the available studies that address the question of interest

Critically low

- *More than one critical flaw with or without non-critical weaknesses*: the review has more than one critical flaw and should not be relied on to provide an accurate and comprehensive summary of the available studies

*Multiple non-critical weaknesses may diminish confidence in the review and it may be appropriate to move the overall appraisal down from moderate to low confidence
